# Patient safety awareness among postgraduate students and nurses in a tertiary health care facility

**DOI:** 10.12669/pjms.335.13780

**Published:** 2017

**Authors:** Attia Bari, Uzma Jabeen, Iqbal Bano, Ahsan Waheed Rathore

**Affiliations:** 1 Dr. Attia Bari, MBBS, DCH, MCPS, FCPS (Paediatric Medicine). Associate Professor of Pediatric Medicine, Department of Paediatric Medicine, The Children’s Hospital and The Institute of Child Health, Lahore, Pakistan; 2 Dr. Uzma Jabeen, MBBS, FCPS. Assistant Professor of Pediatric Medicine, Department of Paediatric Medicine, The Children’s Hospital and The Institute of Child Health, Lahore, Pakistan; 3 Dr. Iqbal Bano, MBBS, FCPS (Paediatric Medicine). Associate Professor of Pediatric Medicine, Department of Paediatric Medicine, The Children’s Hospital and The Institute of Child Health, Lahore, Pakistan; 4 Dr. Ahsan Waheed Rathore, MBBS, DCH, MRCP, MRCPCH, FRCP. Professor of Pediatric Medicine, Department of Paediatric Medicine, The Children’s Hospital and The Institute of Child Health, Lahore, Pakistan

**Keywords:** Medical error, Medical error disclosure, Nurses, Patient safety, Resident

## Abstract

**Objective:**

To determine the knowledge of patient safety among postgraduate residents (PGR) and nurses in a tertiary care hospital.

**Methods:**

This casual comparative study was conducted among the postgraduate residents and nurses working at The Children’s Hospital Lahore in the month of July, August 2017. Both PGR and nurses were asked to complete APSQ-IV questionnaire about patient safety on 7 point Likert scale. Data was analyzed using SPSS version 20 and t-test was used to compare the mean score between two groups. The names of the participants were kept confidential.

**Results:**

A total of 150 residents and 150 nurses were included. The scores of both postgraduate residents and nurses were similar in all domains having positively worded questions with insignificant difference in mean score (p=0.141). In the reverse coded questions the nurses showed positive perception with higher mean score as compared to postgraduate residents (p=0.004). The postgraduate residents in the early years of residency had higher mean score in positively worded question as compared to residents who were in last years of training (p=0.006). There was no difference in the mean score of nurses as regard to their years of experience (p=0.733). Medical error disclosure domain was reported lowest by both postgraduate residents and nurses.

**Conclusion:**

Both postgraduate residents and nurses showed positive attitude with good knowledge and perception towards patient safety. Lowest rated scores were from error disclosure confidence domain.

## INTRODUCTION

Patient safety (PS) and quality improvement of health care delivery to the patients are among the highest priorities of health care system.^1^,^2^ Building a safe health care system means designing processes of care to ensure that patients are safe from accidental injury.^3^ PS education and its training for all health care providers including both doctors and nurses is an important required learning at all levels of training. The literature on PS education in medical school and nursing curricula is not properly developed. Undergraduate medical students and postgraduate trainees still must acquire their PS knowledge through informal education in hospitals because formal training of patient safety curricula is lacking.^4^ A WHO curriculum guide is developed to help medical schools to develop their own patient safety curriculum.^5^

A report on safety in health care by Institute of Medicine publication, To Err is human, focused attention on this problem that every year more Americans die as a result of medical errors than deaths from automobile accidents. There were up to 98,000 deaths per year because of medical errors.^6^ Patients are injured by medical errors in both developed and developing countries. In developing countries, the burden of unsafe care is unclear due to inappropriate infrastructure, insufficient human resources and poorly developed medical error reporting system.^7^ Almost all health care providers have made medical mistakes but they generally don’t tell patients or families about these errors because facing to a medical error is never easy and hence it is not disclosed and are generally underreported.^8^ Health care providers at all training levels experience feelings of guilt and sense of inadequacy of varying degree as a consequence of medical error.^9^

PS is an important topic which must be included in the curricula of both undergraduate postgraduate medical and nursing teaching. In Pakistani medical and nursing school curriculum, there is dearth of information regarding PS and is not given due importance. Postgraduate training period is vulnerable time in which early experience shapes future behavior of residents. Our nurses must also be very familiar with PS. We planned this study to learn about the attitudes and perception of postgraduate residents and nurses about PS.

## METHODS

This was a hospital based cross sectional study, conducted at The Children’s Hospital Lahore which is a tertiary care hospital with 1100 beds and recognized for pediatric postgraduate training. After taking permission via email from the author of Patient Safety Questionnaire her Questionnaire APSQ-IV, a validated instrument was used having 10 domains namely:

1. Patient safety general,
2. Patient safety training received,
3. Error reporting confidence,
4. Error inevitability,
5. Professional incompetence as error cause,
6. Disclosure responsibility,
7. Team functioning,
8. Patient role in error,
9. Importance of patient safety in curriculum
10. Situational awareness.

We used 7 point Likert scales, 7= strongly agree 4=Neutral and 1= strongly disagree (SD) to assess their response. However, ten of 30 items (Q: 3, 13, 14, 15, 16, 17, 19, 24, 26, 28)were negative statements and were scored in reverse order 1=SA and 7=SD. The scoring of individual response to each survey question were classified as a “positive” response if the response was “strongly, agree, agree or somewhat agree) in positively worded questions and (strongly disagree, disagree and somewhat disagree) in reverse coded questions. After receiving approval from the institutional review board, the survey was distributed to a convenience sample comprising 180 post graduate residents (PGR) and 180 staff nurses present on job at the Children’s Hospital Lahore, Pakistan between July and August 2017. Target sample of 150 was achieved with a response rate of 83%. Student nurses, post fellowship senior registrars and consultants working in the hospital were excluded. Data analysis was done by using SPSS version 20.

## RESULTS

The total number of participant included in this study was 300(150 PGR and 150 staff nurses). There was preponderance of female residents 90 (60%) with F:M ratio of 3:2. Most PGR respondents were between 25 and 30 years of age (n=137; 93%) with mean age of 28.19±1.984 and staff nurses having the mean age of 27.31±4.174. PGR belonged to all years of training, (n=66; 44%) were in the first two years of training and (n=95; 63%) of nurses had less than five years of job experience. Details of demographic information is provided in Table-I. The mean APSQ scores, reflecting perception of PGR and nurses are shown in Table-II.

**Table-I:** Characteristics of residents and nurses.

*Category*		*Total n = 150*
Postgraduate residents	*Age*	
	Mean 25-30 years 31-35 years	28.19±1.984 Years 137 (93%) 13 (7%)
	*Sex*	
	F:M Female Male	3:2 90 (60%) 60 (40%)
	*Year of Post Graduate Training*	
	1^st^ Year 2^nd^ Year 3^rd^ Year 4^th^ Year Training complete	44 (29.3%) 22 (14.7%) 39 (26%) 30 (20%) 15 (10%)
Staff nurses	*Age*	
	Mean 21-25 years 26-30 years 31-35 years > 35 years	27.31± 4.174 Years 64 (42.7%) 62 (41.3%) 15 (10.0%) 09 (6.0%)
	*Year of experience*	
	< 5 years 5-10 years >10 years	95 (63.3%) 45 (30%) 10 (6.7%)

**Table-II:** Responses of postgraduate residents’ and nurses to APSQ- IV.

*Domains*	*Questions*	*Postgraduate residents mean score*	*Staff Nurses mean score*
Patient safety: General	When things go wrong, learning from error is more important than disciplining individuals.	5.08 ± 1.859	5.84 ± 1.559
	Patient safety is everyone’s responsibility.	6.16 ± 0.925	6.43 ± 1.107
	Most harm to patients is unavoidable.	4.57 ± 2.198	5.46 ± 2.200
Patient safety training received to date	My training is preparing me to understand the causes of medical errors.	4.69 ± 1.921	5.77 ± 1.898
	I have a good understanding of patient safety issues as a result of my training.	5.28 ± 1.529	6.56 ± 1.000
	My training is preparing me to prevent medical errors.	5.36 ± 1.602	6.15 ± 1.540
Error reporting confidence	I would feel comfortable reporting any errors I had made, no matter how serious the outcome had been for the patient.	4.67 ± 1.820	4.95 ± 2.001
	I would feel comfortable reporting any errors other people had made, no matter how serious the outcome had been for the patient.	4.40 ± 1.630	4.69 ± 1.990
	I am confident I could talk openly to my supervisor about an error I had made if it had resulted in potential or actual harm to my patient.	5.11 ± 1.598	5.68 ± 1.724
Error inevitability	Very experienced health professionals make errors.	5.12 ± 1.524	4.09 ± 2.222
	The clinical environment can cause errors.	5.07 ± 1.688	4.65 ± 1.904
	Human error is inevitable.	5.20 ± 1.622	5.32 ± 1.727
Professional incompetence as error cause	Most medical errors result from careless health professionals.	4.66 ± 1.793	5.40 ± 1.888
	If people paid more attention at work, medical errors would be avoided.	5.61 ± 1.336	6.32 ± 1.462
	Medical errors are a sign of incompetence.	3.93 ± 1.941	5.50 ± 1.831
Disclosure responsibility	It is not necessary to report errors which do not result in harm for the patient.	3.65 ± 1.868	3.09 ± 2.139
	Doctors have a responsibility to disclose errors to patients when they result in harm.	4.37 ± 1.708	4.87 ± 1.982
	All medical errors should be reported	4.93 ± 1.678	6.02 ±1.808
Team functioning	All medical errors should be reported	5.05 ± 5.087	6.04 ± 1.605
	Junior members of a team should think carefully before speaking up about patient safety.	5.66 ± 1.492	5.85 ± 1.496
	For optimum safety, cooperation and sharing of information is crucial.	5.79 ± 1.420	6.22 ± 1.510
Patient’s role in error	Patients have an important role in preventing medical errors.	4.58 ± 1.762	5.13 ± 2.112
	Actively seeking feedback from patients about quality and safety of care is important for patient safety.	5.33 ± 1.446	5.33 ± 2.200
	Patients are not really aware of how safe their care is.	4.97 ± 1.524	5.16 ± 1.980
Importance of patient safety in the curriculum	Teaching students about patient safety should be an important priority in training undergraduates.	5.85 ± 1.395	5.91 ± 1.700
	Patient safety issues cannot be taught and can only be learned through clinical experience when qualified.	4.24 ± 1.948	4.59 ± 2.335
	Learning about patient safety issues before I qualify will enable me to become a more effective health professional.	5.41 ± 1.439	5.59 ± 1.765
Situational awareness	Being on the look-out for potential risks can be detrimental for patient safety.	4.78 ± 1.596	5.61 ± 1.678
	Planning together to deal with problems that may arise is important for patient safety.	5.87 ± 1.166	6.17 ± 1.552
	Understanding the roles and responsibilities of every member of the team is important for patient safety.	5.84 ± 1.419	6.37 ± 1.585

The best scores by PGRs were given for patient safety is every one’s responsibility (6.16) followed by planning together to deal with a problem (5.87) and teaching students about patient safety (5.85). Similarly, the best scores by nurses were given to “good understanding of patient safety issues as a result of my training” (6.56) followed by “understanding the roles and responsibilities of every member of the team is important for patient safety” (6.37).

The lowest rated score was from error reporting confidence domain (question number. 8: “I would feel comfortable reporting any errors other people had made, no matter how serious the outcome had been for the patient”) 4.40 and 4.69 by PGRs and nurses respectively (Table-II).

The scores of both postgraduate residents and nurses were similar in all domains having positively worded questions with insignificant difference in mean score (p=0.141). In reverse coded questions nurses showed higher mean score as compared to PGR (p=0.004) (Table-III). The postgraduate residents in the early years of residency had higher mean score in positively worded question as compared to residents who were in last years of training (p=0.006). There was no difference in the mean score of nurses as regard to their years of experience (p=0.733).

**Table-III:** Comparing mean scores (APSQ- IV) of postgraduate residents’ and nurses.

			*Levene’s Test for Equality of Variances*		*t-test for Equality of Means*						

			*F*	*Sig*	*t*	*df*	*Sig. (2-tailed)*	*Mean Difference*	*Std. Error Difference*	*95% Confidence Interval of the difference*	

										Lower	Upper
Positively worded Questions (Q1,2, 4, 5, 6, 7, 8, 9, 10, 11, 12, 18, 19, 21, 22, 23, 25, 27, 29, 30)	*Mean score/SD* PGR 5.2311± 0.73794 Nurses 5.6459± 0.88015	Equal variance assumed	2.176	0.141	-4.414	298	0.000	-0.41395	0.09378	-0.59850	-0.22939
		Equal variances not assumed			-4.414	298.201	0.000	-0.41395	0.09378	-0.59853	-0.22937
Reverse coded Questions (Q:3,13,14,15,16,17, 20, 24, 26, 28)	*Mean score* PGR 4.7553±0.77623 Nurses 5.4170± 1.08393	Equal variance assumed	8.478	0.004	-6.079	298	0.000	-0.66176	0.10886	-0.87598	-0.44754
		Equal variances not assumed			-6.079	270.002	0.000	-0.66176	0.10886	-0.87607	-0.44745

## DISCUSSION

A global concern is unsafe patient care resulting in 10% of adverse events in hospitalized patients worldwide. This unsafe medical care deserves attention and is a challenging task to improve patient care.^10^ In developing countries due to severe under funding and lack of technologies in health care there are limited interventions available for patient safety.^11^ This study systematically assessed attitudes and perception of PGR and nurses about patient safety and has two important findings. First that both of these groups were in general agreement in all 10 domains measured in the survey. Second in few domains the APSQ score for the PGR were lower than nurses. As far as the knowledge about PS is concerned, the PGR and nurses had almost similar perception except in “error disclosure”. A research done by Mahdeih showed that general perception about patient safety culture was positively reported by 65% of participating nurses.^3^

The 67% PGR in first two years of training agreed in the domains of “patient training to date” than their counterpart PGR who have completed their training 53% (p= 0.001). The findings are not surprising as the differences can be due to their changing roles and responsibilities as PGR progress in their training and there is more direct supervision in initial years of training. This increased supervision to PGR in initial years of training provides additional opportunity for feedback from seniors about their performance and patient safety related issues. Similarly, nurses who had <5 years of experience 84% as compared to those having > 10 years of experience 63% agreed that “my training is preparing me to understand the cause of errors” (p=0.01).

In error inevitability domain, a question “Medical errors are inevitable” both PGR and nurses agreed that “very experienced persons make medical error” (mean score=5.12). A study from Iran and Karachi also showed similar results in which 60% of students agreed with this statement and study by Yahia showed similar results with a mean score of 5.9.^7^,^12^,^13^

A reverse coded question about PS education “Patient safety issues cannot be taught and can only be learned through clinical experience when qualified” was scored in disagreed range both by PGR and nurses consistent with studies done on students perception which showed 80% and 87% of students required education on patient safety topics particularly in disclosing the error to patient and 90% said that they want teaching in analyzing a case to find the cause of medical error.^7^,^14^ A lack of formal teaching practice may result in unsatisfactory error reporting and reluctance to adopt patient safety practices. Various studies focus on the findings that highlight the importance of developing teaching and assessing strategies that explicitly focus on PS issues.^15^,^16^

Both PGR and nurses were less positive about disclosure in error reporting confidence domain as mean score was 4.67±1.820, and 4.95±2.001respectively but still quite positive towards reporting medical errors to their seniors. The two reverse coded questions in the disclosure responsibility domain were appropriately disagreed by both groups but PGR had less score of reporting all medical errors 4.93±1.678 as compared to nurses who had a much higher score 6.02±1.808. Our results are comparable to the results of a qualitative research in which GPs held a positive attitude towards quality improvement through analysis of adverse events and were ready to report such events locally or regionally provided that they are protected from risk exposure to public contempt or to sanctions.^17^ A study published in Medical teachers showed even less mean score of 3.79±1.25 in error disclosure domain.^18^ Studies published in BMC Health Services Research and study done by Maha reported that doctors worry that mistakes made by them are kept in their personal files.^19^,^20^ A research from Iran highlighted the issue of lack of statistics on the incidence of medical errors.^7^ There is also knowledge gaps on what is considered as reportable patient safety event.^21^ Discussing errors with seniors or supervisors had a positive attitude shown by higher mean score in our study. Similar results by various other researches showed that disclosing mistakes may help physicians to learn.^22^,^23^

The biggest challenges in changing the patient safety culture and moving towards a safe health care system is a change from blaming people for errors to one in which errors are taken as opportunity to improve the system and prevent harm to patients. To improve patient safety, PS culture assessment is used to determine targets for interventions and conduct benchmarking.^24^

### Strengths and Limitations

Our study has several strengths and limitations. The strength is that it analyzed the knowledge of PS among both PGR and nurses. Secondly we used a validated and a reliable instrument (APSQ version IV) to measure PS knowledge. Thirdly a much higher response rate was there i.e. 83%. The limitation of our study is that the sample is taken from a single institute so the difference in PGR and nurses from different institutes cannot be sought and this can limit the generalization of our results.

### Implications

This study results can help our leader and curricular developers to include error disclosure teaching as a compulsory part of medical education both in medicine and nursing curricula. PS programs exposure can lead to better PS knowledge and ultimately to better patient care.

## CONCLUSION

A clear positive knowledge of health care professionals of our institute (PGR and nurses)is shown towards patient safety analyzed through this questionnaire. The results encourage pilot projects with the ultimate goal of establishing a feasible reporting system about medical errors.

### Authors Contribution

**AB:** Main author, conceived idea, data collection postgraduate residents, writing of manuscript.

**UJ:** Data collection from nurses, review.

**IB:** Data collection postgraduate residents, review, suggestions.

**AWR:** Final approval, suggestions.
